# Spontaneous termination of chaotic spiral wave dynamics in human cardiac ion channel models

**DOI:** 10.1371/journal.pone.0221401

**Published:** 2019-08-28

**Authors:** Marcel Aron, Sebastian Herzog, Ulrich Parlitz, Stefan Luther, Thomas Lilienkamp

**Affiliations:** 1 Max Planck Institute for Dynamics and Self-Organization, Göttingen, Germany; 2 Institut für Dynamik komplexer Systeme, Georg-August-Universität Göttingen, Göttingen, Germany; 3 Third Institute of Physics, Universität Göttingen, Friedrich-Hund Platz 1, Göttingen, Germany; 4 Bernstein Center for Computational Neuroscience, Friedrich-Hund Platz 1, Göttingen, Germany; 5 DZHK (German Centre for Cardiovascular Research), Partner Site Göttingen, Göttingen, Germany; 6 Department of Pharmacology and Toxicology, University Medical Center Göttingen, Göttingen, Germany; 7 Department of Cardiology and Pneumology, University Medical Center Göttingen, Göttingen, Germany; Georgia State University, UNITED STATES

## Abstract

Chaotic spiral or scroll wave dynamics can be found in diverse systems. In cardiac dynamics, spiral or scroll waves of electrical excitation determine the dynamics during life-threatening arrhythmias like ventricular fibrillation. In numerical studies it was found that chaotic episodes of spiral and scroll waves can be transient, thus they terminate spontaneously. We show in this study that this behavior can also be observed using models which describe the ion channel dynamics of human cardiomyocytes (Bueno-Orovio-Cherry-Fenton model and the Ten Tusscher-Noble-Noble-Panfilov model). For both models we find that the average lifetime of the chaotic transients grows exponentially with the system size. With this behavior, we classify the systems into the group of type-II supertransients. We observe a significant difference of the breakup behavior between the models, which results in a distinct dynamics during the final phase just before the termination. The observation of a (temporally) stable single-spiral state affects the prevailing description of the dynamics of type-II supertransients as being “quasi-stationary” and also the feasibility of predicting the spontaneous termination of the spiral wave dynamics. In the long term, the relation between the breakup behavior of spiral waves and properties of chaotic transients like predictability or average transient lifetime may contribute to an improved understanding and classification of cardiac arrhythmias.

## Introduction

Transient chaos is a widespread phenomenon, where the chaotic dynamics of a system is not persistent but decays after some time. Chaotic transients appear in diverse systems [1] as, for instance, in ecology [2], turbulence [3], coupled semiconductor oscillators [4], neural networks [5–7] or NMR-lasers [8]. Regarding the control of such chaotic transients it depends on the specific system or objective whether the chaotic phase should be extended or a quick self-termination is desired. An example for the latter case can be found in the field of cardiac dynamics, where chaotic spiral or scroll waves determine the electrical excitation dynamics during life-threatening cardiac arrhythmias like ventricular fibrillation [9–12]. Spontaneous termination of such ventricular fibrillation in human patients has been reported [13] and it has been discussed how the underlying mechanisms of self-terminating arrhythmias could improve the understanding and efficiency of antiarrhtyhmic drugs [14, 15].

Also, in numerical simulations it was found that chaotic spiral wave dynamics can in fact be transient [16–18]. However, it was not yet clear whether the results hold also for human cardiac ion channel models. Furthermore, former studies showed that chaotic transients of spiral and scroll wave dynamics observed in excitable media can be assigned to the group of type-II supertransients, which are characterized by a spontaneous self-termination, which is, so far, not predictable a significant amount of time ahead. Although it has been shown that the state space structure itself changes nearby the “exits” of the chaotic regime [19, 20], precursors based on conventional variables have not been found yet.

In this study we show that the transient nature of chaotic spiral wave dynamics is a robust phenomenon which holds also for human cardiac ion channel models, explicitly the Bueno-Orovio-Cherry-Fenton model [21] and the Ten Tusscher-Noble-Noble-Panfilov model [22, 23]. Furthermore, we reveal that the wave breakup behavior of the dynamics can have a large impact on the mechanism that leads to self-termination, and in this way may have implications for a possible prediction of self-termination of spiral wave dynamics, which we observe during ventricular fibrillation.

This study is organized in the following way: In the next section we present the investigated models and describe how we determined average transient lifetimes 〈*T*〉 in those systems. In the next section we demonstrate that the average lifetimes increase exponentially with the system size in both models, whereas the number of spiral waves increases linearly. Then, we investigate how the differences of spiral wave breakup behavior between both models can affect the final episode of the transients. In the last section, we discuss how these findings can contribute towards an improved understanding of the underlying mechanisms of spiral wave chaos, which occurs, for instance, during ventricular fibrillation and how future simulations of cardiac arrhythmias could benefit from these results.

## Materials and methods

We investigate two models of cardiac ion channel dynamics in a two-dimensional squared shaped domain with the domain size *A* = *L*_x_ × *L*_y_, where *L*_i_ is the length of the domain in direction “i”. The underlying reaction-diffusion equations of both models will be explained in the next section. The creation of initial conditions and the detection of phase singularities is discussed in the following section. Further details about both methods and a detailed description of the algorithms are given in S1 Appendix.

### The system of reaction-diffusion equations

The propagation of electrical excitation waves through cardiac tissue was modeled by a system of reaction-diffusion equations describing the evolution of the transmembrane potential *V*_m_:

$$\begin{array}{c} \partial_{t}V_{m}=\nabla \cdot (D\nabla V_{m})-\left(I_{\text{ion}}+I_{\text{stim}}\right)/C_{m}, \end{array}$$ (1)

where *C*_m_ is the membrane capacitance, *D* the diffusion tensor (in our case isotropic), *I*_ion_ the net transmembrane ion current, and *I*_stim_ an external stimulation current, which is used for the creation of initial conditions only (details about the creation of initial conditions are given later). The individual transmembrane currents within *I*_ion_ as well as additional ion channel dynamics constitute the remaining body of differential equations. In this study we investigated the Bueno-Orovio-Cherry-Fenton model (from now on denoted as the BOCF model) and the Ten Tusscher-Noble-Noble-Panfilov model [22] with the update of the original model regarding the description of the calcium dynamics, as discussed in [23] (from now on denoted as the TNNP model).

The Laplacian (first term on the right-hand side of Eq (1)) was discretized using a nine-point stencil. The temporal integration was performed through a first-order explicit Euler step for all equations except those pertaining to the dynamics of gating variables, which were evolved through a first-order Rush-Larsen scheme instead [24]. Most computations were carried out on a cluster of 32 Nvidia Tesla P100 GPUs with 16 GB VRAM and a 32-bit floating-point unit performance of 10 TFLOPS each.

#### The Bueno-Orovio-Cherry-Fenton model

Instead of modeling the large variety of distinct ion channel currents present in a cardiac cardiomyocyte, the Bueno-Orovio-Cherry-Fenton model aims at describing the net transmembrane current by three contributions: a fast inward current *I*_fi_, slow inward current *I*_si_, and slow outward current *I*_so_. Due to this “effective” description of the dynamics, it is convenient to introduce the dimensionless membrane potential *u* = (*V*_m_ − *V*_rest_)/(*V*_fi_ − *V*_rest_) which is normalized by the resting membrane potential *V*_rest_ and some upper limit, e.g. the Nernst potential of the fast inward current *V*_fi_. Hence similarly rescaled currents can be introduced by, for instance, *J*_fi_ = *I*_fi_/(*C*_m_(*V*_fi_ − *V*_rest_)). Using this rescaling Eq (1) becomes with the three “effective” currents *J*_fi_, *J*_so_, and *J*_si_:

$$\begin{array}{c} \partial_{t}u=\nabla \cdot (D\nabla u)-\left(J_{\text{fi}}+J_{\text{so}}+J_{\text{si}}+J_{\text{stim}}\right)\;, \end{array}$$ (2)

where the rescaled currents have the unit of inverse time. *J*_stim_ indicates the (rescaled) external stimulation current, which is used for the creation of initial conditions.

In the Bueno-Orovio-Cherry-Fenton model (BOCF model) the ionic currents are described by

$$J_{\text{fi}}=-v\;H\left(u-\theta_{v}\right)\;\left(u-\theta_{v}\right)\;\left(u_{u}-u\right)/\tau_{\text{fi}}\;,$$ (3)

$$J_{\text{so}}=\left(u-u_{o}\right)\;\left(1-H\left(u-\theta_{w}\right)\right)/\tau_{o}+H\left(u-\theta_{w}\right)/\tau_{\text{so}}\;,$$ (4)

$$J_{\text{si}}=-H\left(u-\theta_{w}\right)\;w\;s/\tau_{\text{si}}\;,$$ (5)

where *v*, *w*, and *s* are gating variables, *θ*_*v*_, *u*_*u*_, *u*_o_, and *θ*_*w*_ model parameters, and *τ*_fi_, *τ*_o_, *τ*_so_, and *τ*_si_ time constants.

**Fig 1:**
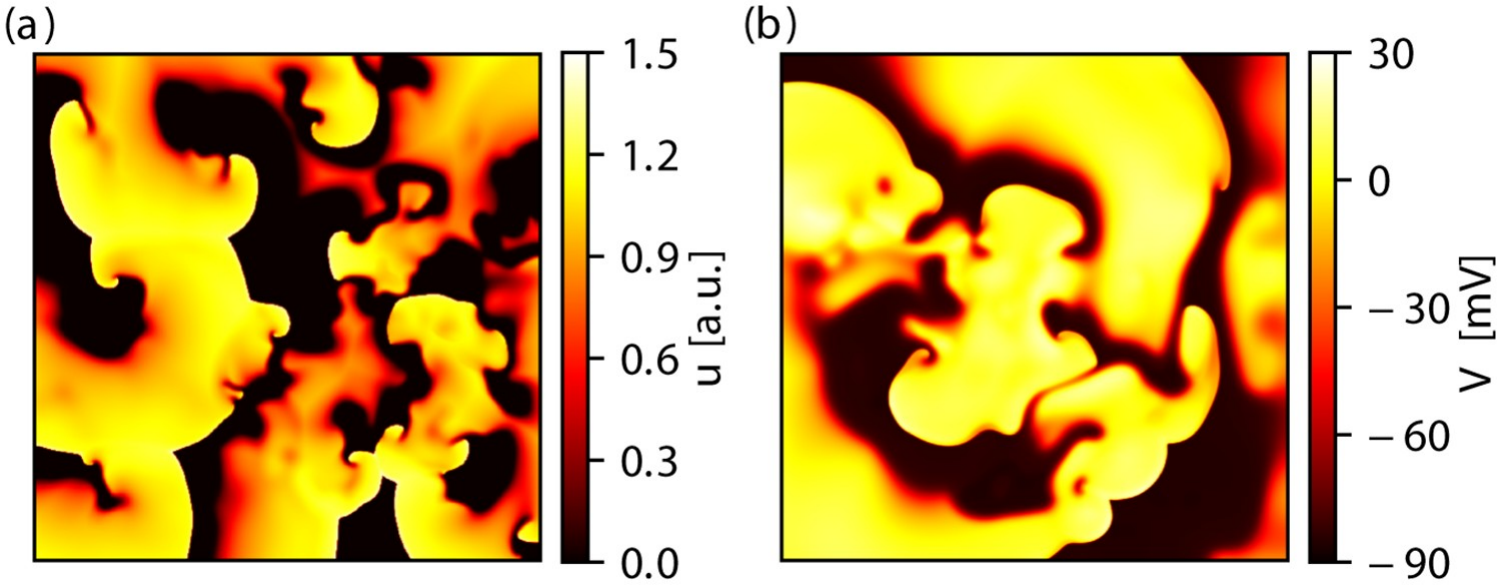
Exemplary snapshots of the membrane potential of chaotic spiral wave dynamics in two-dimensional simulations. The rescaled membrane potential *u* is shown for the Bueno-Orovio-Cherry-Fenton model in subplot (a)(domain size of *A* = 2.62 × 10^5^ mm^2^). In the case of the Ten Tusscher-Noble-Noble-Panfilov model *V*_m_ is shown in subplot (b) (domain size of *A* = 0.66 × 10^5^ mm^2^).

A value of *D* = 2 × 10^2^ mm^2^/s was selected for the isotropic diffusion tensor. The model parameters chosen correspond to the “TNNP” parameter set in [21], thus the parameter set aims at reproducing the dynamics of the original TNNP model [22]. For the numerical integration scheme, a spatial resolution of *h* = 1 mm along with a time step of length Δ*t* = 1 × 10^−4^ s was used. A snapshot of the normalized membrane potential *u* during a typical episode of spiral wave chaos is shown in Fig 1(a).

#### The ten Tusscher-Noble-Noble-Panfilov model

The revision of the Tusscher-Noble-Noble-Panfilov model (TNNP model) was used for all computations [23]. It features twelve transmembrane currents and six state variables governing the temporal evolution of ion concentrations. The model was implemented based on the description by the CellML project [25] with parameters mostly taken from the epicardial parameter set from [23] with the exception of *C*_m_ = 0.2 × 10^−2^ *μ*F/mm^2^ and *D* = 1.54 × 10^2^ mm^2^/s (isotropic). The numerical computations were carried out with a spatial resolution of *h* = 0.2 mm and a time step of length Δ*t* = 2 × 10^−5^ s. Additionally, the “slope 1.8” modifications in [23] were applied to enable spiral wave breakup. A snapshot of the membrane potential *V*_m_ during a typical episode of spiral wave chaos is shown in Fig 1(b).

### Creation of initial conditions and detection of phase singularities

Since relevant quantities discussed in this study vary during a single simulation (e.g. the number of spiral waves) or between different initial conditions (e.g. the transient lifetime), we determined mainly averaged quantities. In order to provide statistically robust statements, it is necessary to create many different initial conditions which sufficiently sample the relevant region of the state space. To create such a set of independent initial conditions, we used a sequence of spatially distributed local stimuli. Details about this process can be found in the Appendix. An exemplary video of the creation of an initial condition of the BOCF model is given as supporting information (S1 Video).

The number of spiral waves was determined via detecting the phase singularities which are present in the system. This procedure was done by a two-step protocol: In a first step, the phase *θ* was determined by a two-dimensional phase-space projection. Phase singularities (which can be associated with the tips of the spiral waves) could then be identified at a specific position by evaluating closed line integrals of the gradient of *θ* encompassing the spot in space. Further details about the procedure can be found in the Appendix.

### Determination of the average transient lifetime

**Fig 2:**
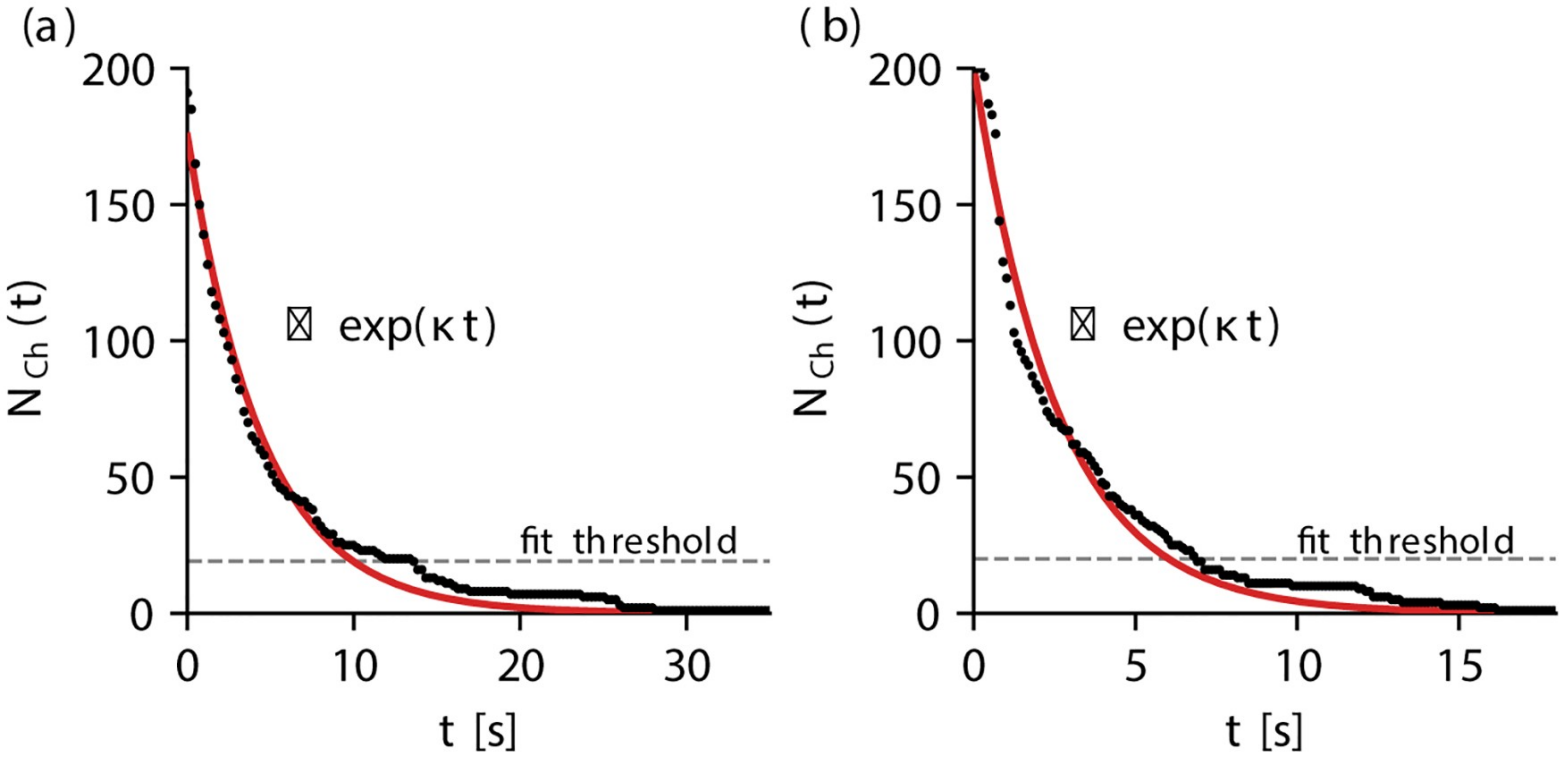
Exemplary temporal evolutions of the number of chaotic initial conditions *N*_Ch_(*t*) which show chaotic dynamics at time *t*. In subplot (a) and (b) *N*_Ch_(*t*) is shown for the BOCF model (domain size of *A* = 0.66 × 10^5^ mm^2^) and the TNNP model (domain size of *A* = 0.32 × 10^5^ mm^2^), respectively. For both models, 200 initial conditions were created for each domain size. The red lines indicate the fits of the data according to Eq (6), where data points below the fit threshold were discarded.

We quantify chaotic transients by determining their average lifetimes which correspond to a specific domain size *A*. In a system exhibiting transient chaos, the number of initial conditions with a transient lifetime of at least *t* from a sample of size *N*, *N*_Ch_(*t*), is expected to decay exponentially [1]:

$$\begin{array}{c} N_{\text{Ch}}\left(t\right)∼\exp\left(-\kappa \;t\right)\;, \end{array}$$ (6)

where *κ* denotes the escape rate describing the “escape” from the chaotic regime of the state space which is usually determined by a chaotic saddle, towards the non-chaotic region. In our case, the latter can be identified as the global resting state of the system without excitation wave dynamics. Thus, the escape rate counts the number of initial conditions which leave the chaotic regime of the state space. It can be related to the system’s average transient lifetime by *κ*^−1^ ≈ 〈*T*〉 [1], where the approximate equality is due to the specific realization of a finite number of initial conditions in the state space. This relationship was used to determine the average transient lifetime of both models for various domain sizes by creating 200 random initial conditions for each model/size configuration. Subsequently, a non-linear least-squares regression in line with Eq (6) was performed, and the escape rate (respectively the average lifetime) was extracted. *N*_Ch_(*t*) is exemplarily shown for both models in Fig 2(a) and 2(b) for the BOCF model (domain size of *A* = 0.66 × 10^5^ mm^2^) and the TNNP model (domain size of *A* = 0.32 × 10^5^ mm^2^), respectively. The fit was performed for values of $N_{\text{Ch}}\left(t\right)>\text{fit}\;\text{threshold}$ (dashed gray lines in Fig 2) which was chosen as fit threshold = 20 (10% of the number of created initial conditions) in order to neglect a huge influence of the tail of the exponential where statistics was poor.

## Results

The presented results are structured in the following way: In the first section, we discuss how the average lifetime of chaotic spiral wave episodes depends on the system size. In the following part, we show that the average number of spiral waves grows linearly with the system size. We then demonstrate how the number of spiral waves is distributed differently in both investigated models, respectively, and how this has an impact on the correlation between the average number of spiral waves and the end of the chaotic episode (last section).

### Transient lifetime and average spiral wave number depend on the system size

The spiral wave dynamics we observe in simulations of both models is transient, meaning that after a certain amount of time no more spiral waves are present and the system returns globally to the resting state. During the chaotic episode, a fluctuating number of spiral waves can be observed, thus spiral waves are created/annihilated permanently until no spiral waves are left. A representative episode of transient spiral wave chaos of the TNNP model is given as supporting information (S2 Video).

**Fig 3:**
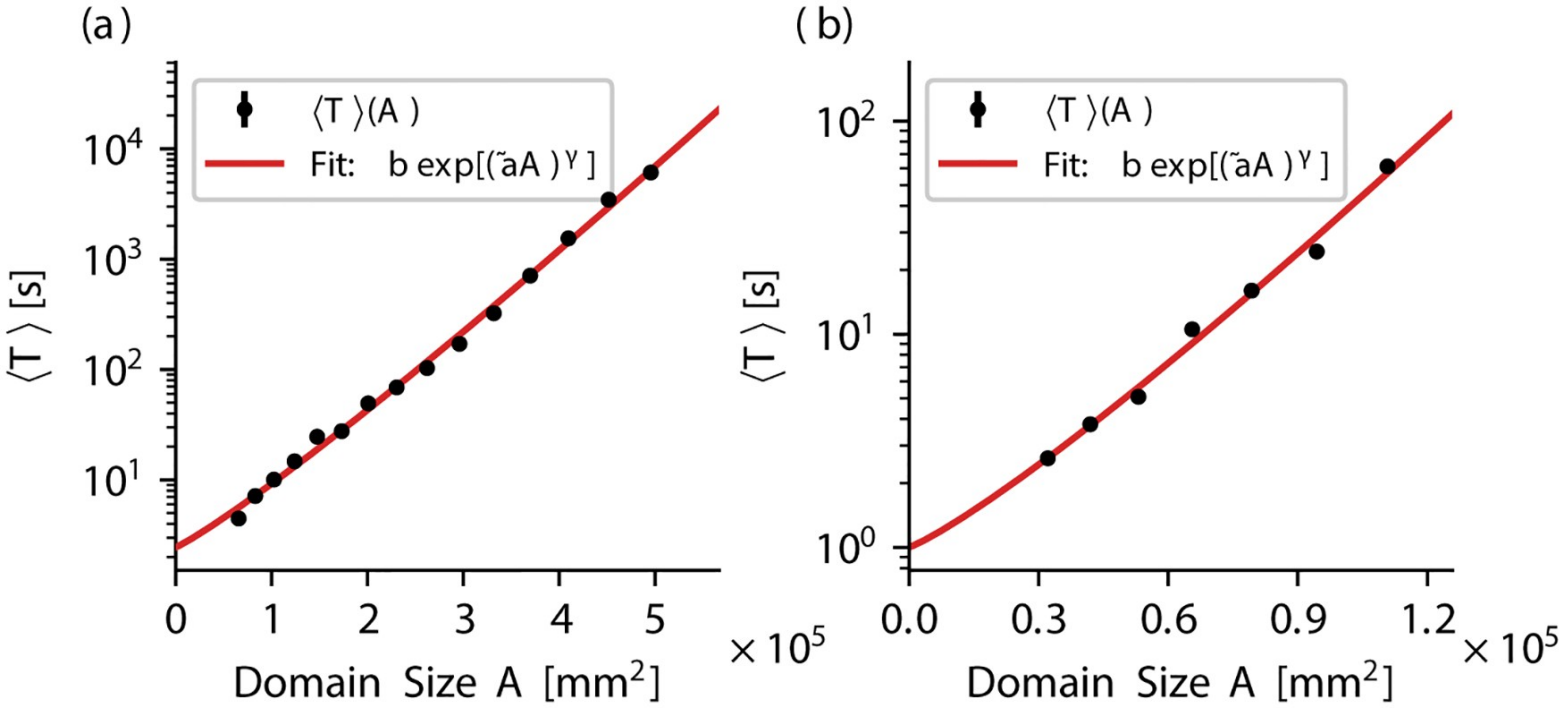
The exponential scaling of 〈*T*〉. For both models, the average transient lifetime 〈*T*〉 is shown for various domain sizes *A* in subplot (a) and (b), respectively. The red line in both subplots shows a fit of the data according to Eq (7), which indicates the exponential scaling of the average transient lifetime with the system size. The corresponding fit parameters are given in Table 1. The numerical data underlying this graph and the following figures are also provided as Supporting information S1 Data.

We characterize the transient chaotic spiral wave episodes in both models by determining the corresponding average lifetime. For this purpose, 200 initial conditions were created and their average lifetime was determined (details can be found in the methods section). This procedure was realized for varying system size domains. Fig 3(a) and 3(b) show an exponential increase of the average lifetime 〈*T*〉 with the system size *L* for the BOCF model and the TNNP model, respectively.

Spatially extended systems with transient dynamics whose lifetime grows rapidly with the system size are called “supertransients”. More specifically, the group of type-II supertransients are characterized by an exponential scaling behavior of the lifetime of the transients with the system size [26]:

$$\begin{array}{c} \left\langle T\right\rangle ∼\exp[{\left(\tilde{a}A\right)}^{\gamma }]\;. \end{array}$$ (7)

In Fig 3(a) and 3(b) the data was fitted according to Eq (7) (linear least-squared fit of the natural logarithms of the data) for both models (solid red lines in Fig 3(a) and 3(b), respectively), and the obtained parameters are given in Table 1.

**Table 1:** Fit parameters $\tilde{a}$ and *γ* according to Eq (7) linear least-squared fit of the natural logarithms of the average lifetimes 〈*T*〉 depicted in Fig 3(a) and 3(b), for the BOCF model and the TNNP model, respectively). The standard deviation was determined based on the variance of each parameter according to the least-squared method.

Model	$\tilde{a}\;\left[1/{\text{mm}}^{2}\right]$	*γ*[a.u.]
BOCF	1.28 × 10^−5^(±1.81 × 10^−6^)	1.11(±7.42 × 10^−2^)
TNNP	3.02 × 10^−5^(±1.20 × 10^−5^)	1.15(±2.84 × 10^−1^)

The spiral waves are the organizing objects which determine the dynamics during chaotic episodes. In particular, the creation and annihilation of spiral waves during the episode is the underlying mechanism for the self-termination in the end. In order to quantify how an increase of the domain size changes the spiral wave dynamics, we furthermore measured the average number of spiral waves for each system size *A* by determining the organizing centers of the spiral waves, so called phase singularities. Details about the process of determination can be found in the supporting information section.

**Fig 4:**
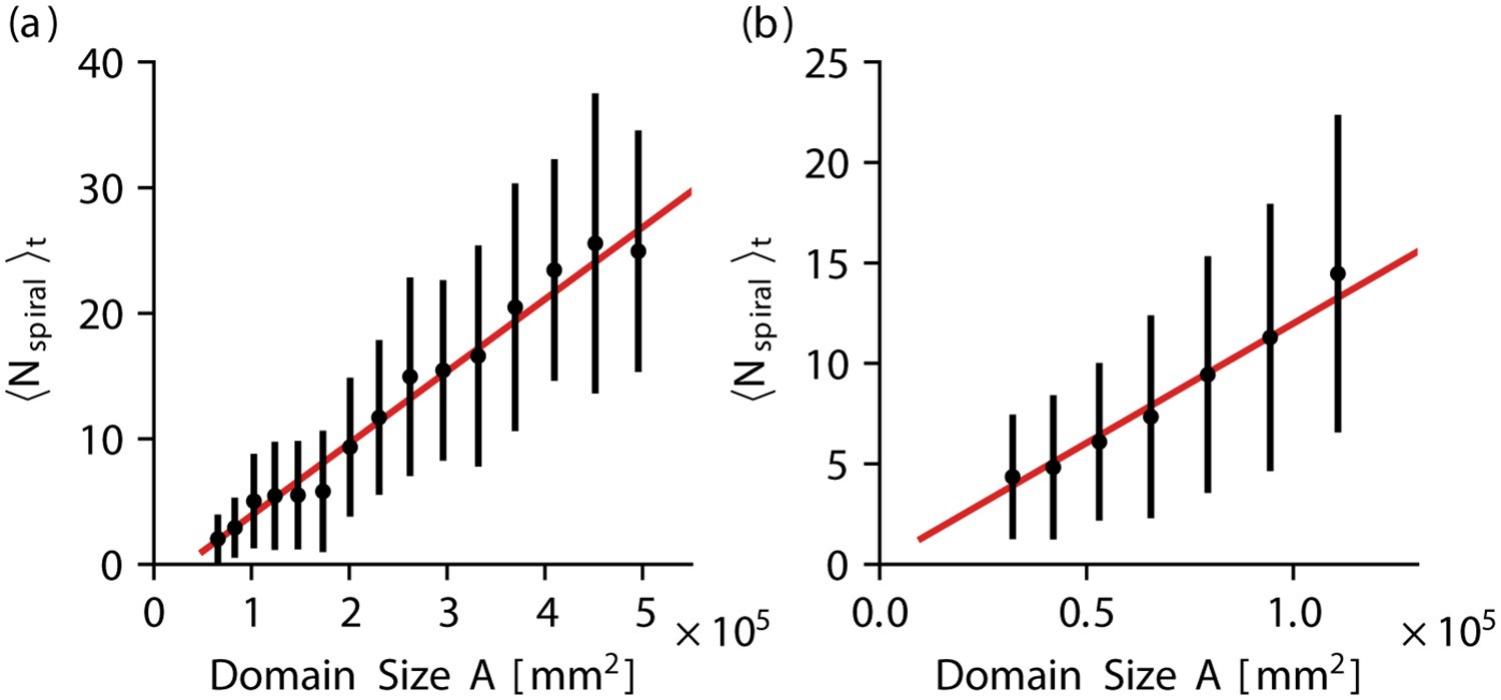
The average number of phase singularities grows with the domain size. The average number of spiral waves 〈*N*_spiral_〉_*t*_ (averaged over simulation time *t*) present in a system is shown for various domain sizes *A* for the BOCF model (a) and the TNNP model (b), respectively. Error bars represent the standard deviation, which is significant since the number of spiral waves usually fluctuates considerably during a typical episode.

In Fig 4(a) and 4(b) the average number of spiral waves (averaged over time) 〈*N*_spiral_〉_*t*_ is shown for various domain sizes for the BOCF model and the TNNP model, respectively. It is noteworthy that the subscript *t* in 〈*N*_spiral_〉_*t*_ indicates that the averaging (denoted by the brackets) is performed over simulation time. The temporal average shown in Fig 4 was determined based on a total simulation time of *T*_obs_ = 80 s for each domain size. Details about the procedure can be found in the Appendix.

Since the number of spiral waves present in the system fluctuates during a typical episode of chaotic spiral wave dynamics, the standard deviation of 〈*N*_spiral_〉_*t*_ indicated by the error bars is rather large. However, a linear increase of the average spiral wave number could be verified in both models (subplots (a) and (b), respectively). This observation is in agreement with similar studies of other ion channel models of transient spiral wave dynamics [16, 17].

### Wave breakup behavior affects final phase of transients

The underlying mechanism which determines the transient spiral wave dynamics is the continuous creation and annihilation of spiral waves, causing a fluctuating number of spiral waves present in the system, which reaches zero at the point in time of self-termination. Whereas spiral waves can annihilate with each other (pair annihilation) or with the boundary (in the case of no-flux boundary conditions), there are also different processes leading to the creation of spiral waves. C. Marcotte and R. Grigoriev [27] distinguish between “wave breakup” and so called “wave coalescence”.

In the first case, the front of an excitation wave interacts with a waveback of the same wave (e.g. induced by a conduction block) and increases the number of excitation waves in this way. “Wave coalescence”, however, is denoted as the interaction of an excitation front with a refractory region associated with another wave and the following creation of two separated wavesfronts. Whereas the first mechanism can considered responsible for the transition from a single-rotor state (which can be associated with tachycardia) to a more chaotic multi-spiral state (which can be associated with fibrillation), the process of “wave coalescence” is rather linked to the maintenance of an already complex state which exhibits many spirals. In fact, numerical studies have shown that when the system is already in a multi-spiral state, “wave coalescence” is the dominant mechanism for the creation of spiral waves [27] (C. Marcotte and R. Grigoriev did not find a single instance of wave breakup in simulations during multi-spiral chaos).

**Fig 5:**
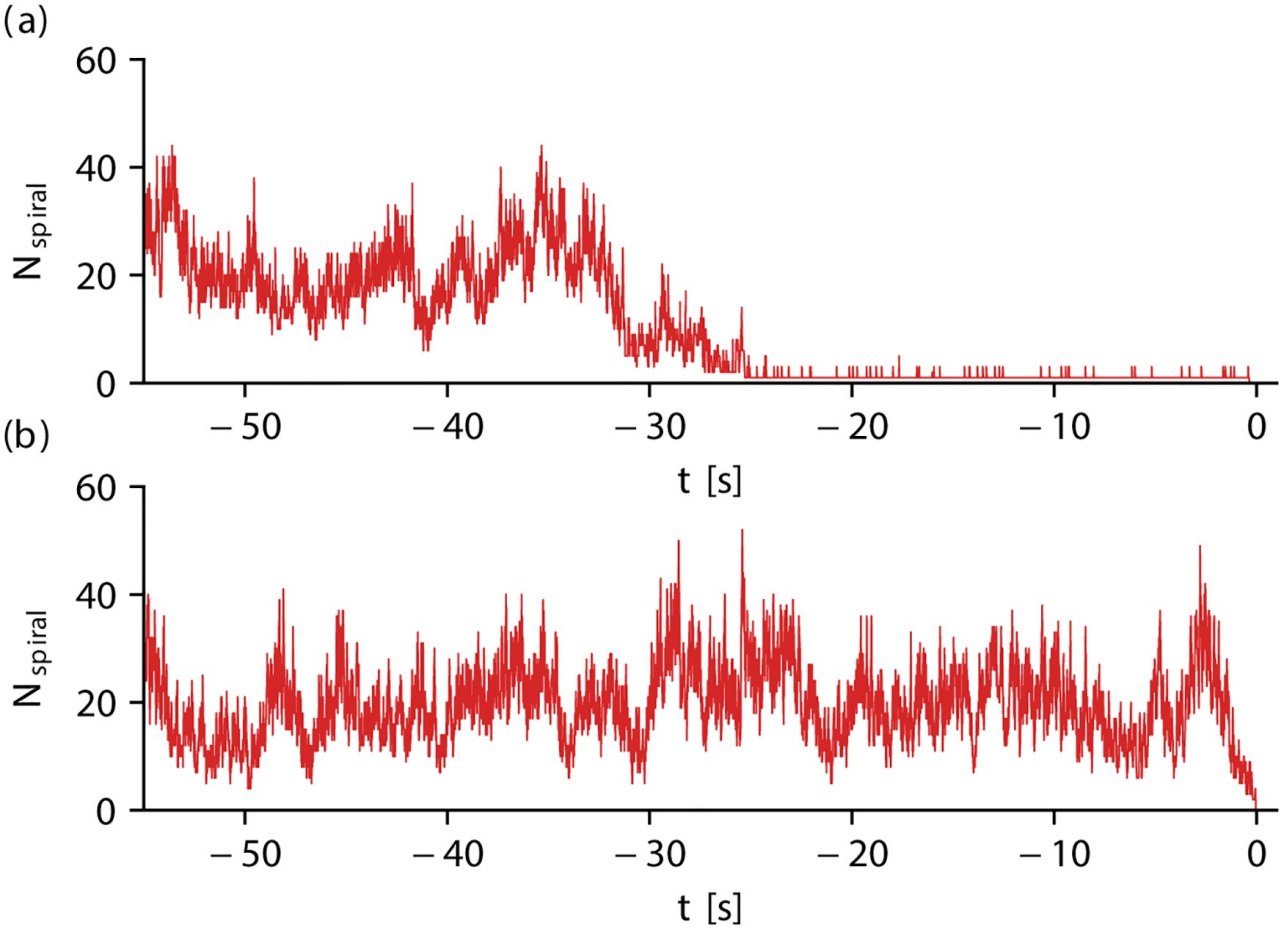
The temporal evolution of the number of spiral waves *N*_spiral_ during typical episodes of transient chaos. The number of spiral waves is shown for a typical example in subplot (a) for the BOCF model (domain size of *A* = 4.96 × 10^5^ mm^2^) and in subplot (b) for the TNNP model (domain size of *A* = 1.11 × 10^5^ mm^2^). In the latter case, the system terminates “spontaneously”, whereas in the case of the BOCF model, the system decays into the single-spiral state (around *t* ≈ −25 s) and terminates much later (around *t* = 0 s).

As also stated in [21] we observe in our simulations that a “wave breakup” occurs only very rarely in the BOCF model, whereas the TNNP model promotes “wave breakup”. However, since we initialize both systems in such a way that several spiral waves are present, the number of spiral waves fluctuates in both models. A qualitative difference can only be observed for states of a low number of spiral waves (in particular *N*_spiral_ = 1). Since wave breakup is not promoted in the BOCF model, the single-spiral state is quite robust and persists for a significant amount of time. This behavior is explicitly relevant during the final phase of the investigated transient episodes. Fig 5(a) and 5(b) show the number of spiral waves *N*_spiral_ for exemplary episodes of chaotic spiral wave dynamics for the BOCF model and the TNNP model, respectively. Time is normalized such that the respective self-termination occurs at *t* = 0. In both cases *N*_spiral_ fluctuates at the beginning of the time series. In Fig 5(a) the system deteriorates into the single-spiral state at around *t* ≈ −25 *s* and returns to the resting state much later. However, the transition to the single-spiral state does not necessarily imply a subsequent self-termination. The evolution of *N*_spiral_ for another episode of the BOCF model is given in S1 Fig, in which the system shows a (temporally) intermediate single-spiral state, but increases its number of spiral waves again afterwards. Exemplary videos of the initial condition discussed in S1 Fig which show the transition from a multi-spiral state into the single-spiral state and the transition back into a multi-spiral state are given as supporting information (S3 and S4 Videos, respectively). In the case of the TNNP model (Fig 5(b)) the general behavior of a pronounced single-spiral state cannot be observed but *N*_spiral_ fluctuates continuously and reaches zero rather “spontaneously” (see Fig 5(b)).

### Quantifying differences of spiral wave dynamics

**Fig 6:**
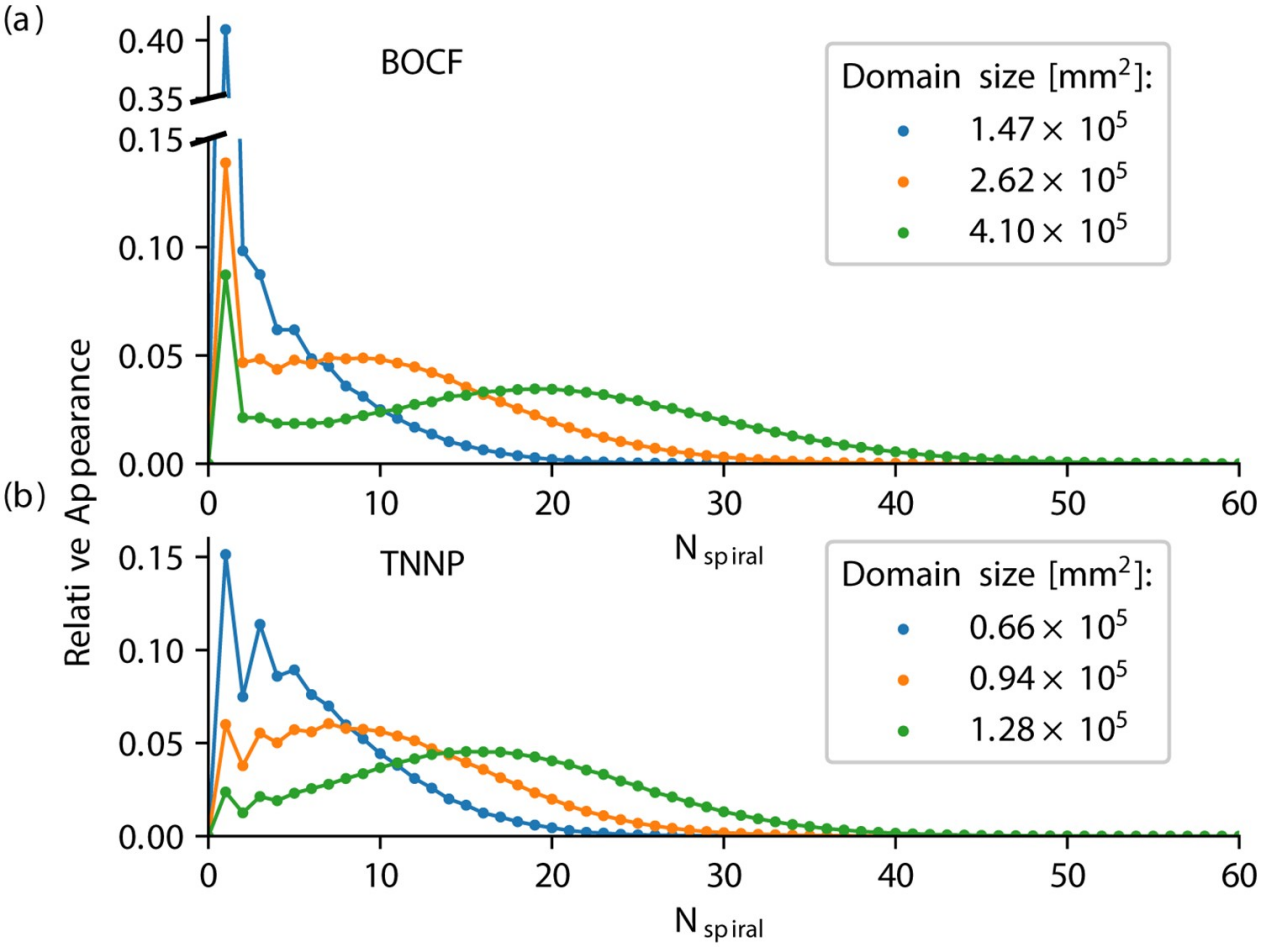
The relative appearance of particular numbers of spiral waves *N*_spiral_ present in the system at the same time. For three different domain sizes each (denoted by different colors) the distribution of the relative appearance of *N*_spiral_ is shown for the BOCF model (a) and the TNNP model (b), respectively. In both cases, the distribution shifts towards larger *N*_spiral_ with increasing domain sizes. The shape is, however, biased for low number of spiral waves. In particular in the case of the BOCF model, a distinct peak for the single-spiral state for all three domain sizes can be observed.

We want to discuss and interpret the quantitative difference of the spiral wave dynamics between the two models shown in the previous section in a more abstract/mathematical description: As stated in [17], the transient dynamics of spiral wave chaos can be described as a Markov chain, where each state is determined by the number of the spiral waves of the system. Transition probabilities determine how likely a state will increase or decrease the number of spiral waves. For both models, we statistically investigated the probability of finding the system in a state with a specific number of spiral waves, sketched in Fig 6(a) and 6(b), for the BOCF model, and the TNNP model, respectively. For both models we measured *N*_spiral_, for three distinct domain sizes for each model, respectively. Except for low numbers of *N*_spiral_, a smooth distribution can be observed, with a local maximum which shifts towards larger *N*_spiral_ for larger domains. However, in the case of the BOCF model the pronounced characteristic of the single-spiral state is clearly visible which is mostly insignificant in the case of the TNNP model (the somewhat non smooth behavior for a domain size of *A* = 0.66 × 10^5^ mm^2^ is caused by a relatively small domain size, where the maximum value of the distribution is close to *N*_spiral_ = 0).

**Fig 7:**
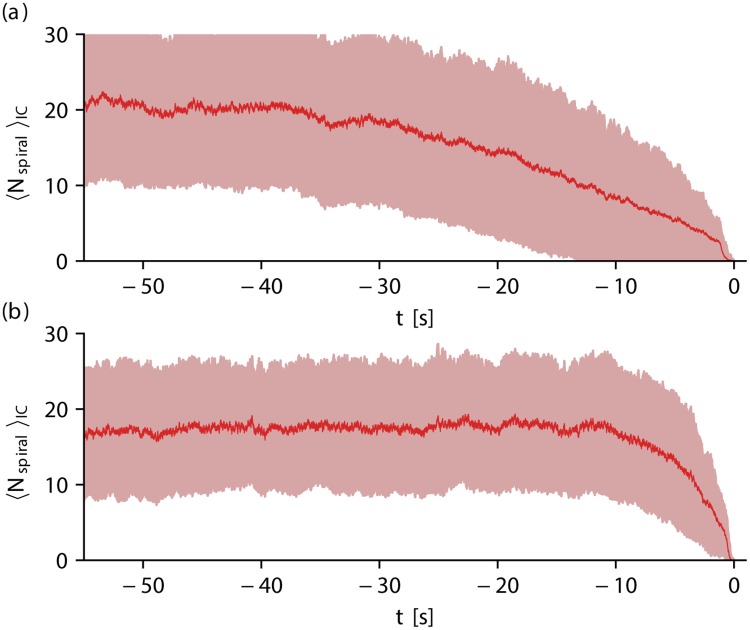
The mean number of spiral waves, averaged over 200 initial conditions 〈*N*_spiral_〉_IC_ is shown for both models. 〈*N*_spiral_〉_IC_ is shown as red solid lines (light red area indicates the standard deviation) in subplot (a) for the BOCF model (domain size of *A* = 4.10 × 10^5^ mm^2^) and in subplot (b) for the TNNP model (domain size of *A* = 1.28 × 10^5^ mm^2^). Initial conditions were normalized in such a way that self-termination occurs at *t* = 0 s. In the latter case, 〈*N*_spiral_〉_IC_ decreases around *t* ≈ −10 s, whereas in the case of the BOCF model, 〈*N*_spiral_〉_IC_ decreases much earlier (around *t* ≈ −40 s).

The former investigations show that the wave breakup behavior of the dynamics can have a significant impact on the terminal phase of self-terminating episodes. We verified this observation by measuring *N*_spiral_ before self-termination for 200 initial conditions for each model, respectively. Fig 7(a) and 7(b) show the number of spiral waves for the BOCF model (domain size of *A* = 4.10 × 10^5^ mm^2^) and the TNNP model (domain size of *A* = 1.28 × 10^5^ mm^2^), respectively, averaged over 200 initial conditions 〈*N*_spiral_〉_IC_, where time was normalized such that all initial conditions terminate at *t* = 0 s. Note that the subscript IC in 〈*N*_spiral_〉_IC_ indicates that the average denoted by the brackets is performed over initial conditions, and not over time (in contrast to 〈*N*_spiral_〉_*t*_ shown in Fig 4, for example).

Far from the point of self-termination, 〈*N*_spiral_〉_IC_ (thick red line) saturates in both cases at values (〈*N*_spiral_〉_IC_ ≈ 20, and 〈*N*_spiral_〉_IC_ ≈ 18, respectively) coinciding with the average number of spiral waves in a system (compare Fig 4(a) and 4(b), respectively). While in the case of the TNNP model (subplot Fig 7(b)) 〈*N*_spiral_〉_IC_ starts to deviate significantly from this value around t≈−10s, the value decreases much earlier (around t≈−40s) in the case of the BOCF model (subplot Fig 7(a)). This significant difference between the models is also a consequence of the pronounced characteristic of the single-spiral state before self-termination present in the BOCF model.

### Wave breakup behavior of transient spiral wave chaos in the context of type-II supertransients

**Fig 8:**
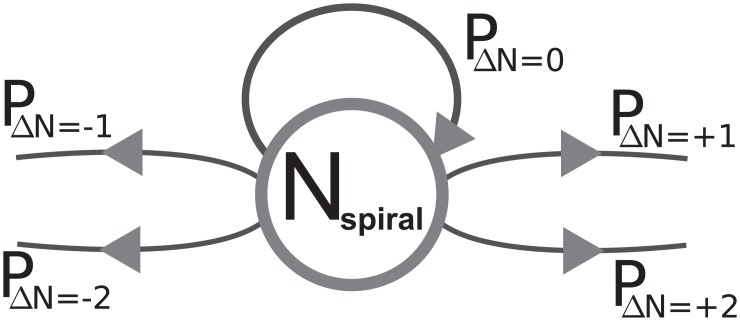
Symbolic Markov chain description of the temporal evolution of spiral wave chaos. Each state of the system can be characterized by the number of spiral waves *N*. When evolving the system over a small but finite amount of time, the number of spiral waves can remain the same (described by the probability *P*_ΔN=0_), or increase/decrease (described by the probabilities *P*_ΔN=+1_, *P*_ΔN=+2_ and *P*_ΔN=−1_, *P*_ΔN=−2_, respectively). When time intervals of the evolution are small enough, the change of *N*_spiral_ is limited by Δ*N* = {±1, ±2}.

With the exponential relation between the system size *A* and the transient lifetime 〈*T*〉 we found the typical scaling behavior of type-II supertransients. In [26] the dynamics of such systems is described as “quasi-stationary” with a spontaneous collapse of the dynamics towards some (low-dimensional) attractor. In the case of chaotic spiral wave chaos, we can typically observe a fluctuating number of spiral waves, and the temporal point of self-termination can not easily be predicted by using “conventional” variables (e.g. *N*_spiral_ in Fig 5(b)). However, the mechanism of self-termination can in the case of spiral wave chaos be described in an ordered way by using the above-mentioned concept of Markov chains [17]: All trajectories have to pass the state of *N*_spiral_ = 1 (*N*_spiral_ = 2) before terminating via collision with the boundary (or via a pair annihilation in the case of *N*_spiral_ = 2 spirals), described by the Markov chain by a transition towards the state with *N*_spiral_ = 0. The transitions from one state to another are characterized here by transition probabilities. The governing processes of creation and annihilation of spiral waves allow only a change of the number of spiral waves by ±1 or ±2 (if considering arbitrarily small time intervals). In general, the temporal evolution of a state with *N* spiral waves from time *t* to *t* + Δ*t* can be described by five probabilities: The system can remain in the same state, meaning the number of spiral waves does not change (*P*_ΔN=0_), or the number of spiral waves can increase, or decrease by different mechanisms (*P*_ΔN=+1_, *P*_ΔN=+2_, or *P*_ΔN=−1_, *P*_ΔN=−2_, respectively). Fig 8 depicts the three possibilities for one exemplary state of the Markov chain. In this way, in each system the number of spiral waves needs to decrease until self-termination can occur. On average this process takes some time, resulting in the decrease of 〈*N*_spiral_〉_IC_ in Fig 7 a significant amount of time before the actual collapse.

Regarding the Markov chain description, changes of the wave breakup behavior of spiral waves can significantly alter these transition probabilities. In particular for transitions from the *N*_spiral_ = 1 state, *P*_ΔN=0_ is much larger compared to transition probabilities with Δ*N* ≠ 0 in the BOCF model, as it is in the case of the TNNP model: Since “wave breakup” of a single spiral wave does occur only rarely in the case of the BOCF model, the system remains in the same state for a significant time. For states with *N*_spiral_ > 1, the number of spiral waves changes much faster (Fig 5(a)) and the transition probabilities with Δ*N* ≠ 0 are increased (due to wave coalescence).

In the context of type-II supertransients, the single-spiral state before self-termination is a distinct state compared to states with *N*_spiral_ > 1 (distinguished by significant variations of transition probabilities and occupation times). This does not imply the existence of a precursor of the upcoming collapse in the classical sense, but the temporally structured course of the self-termination (via the “intermediate” *N*_spiral_ = 1 state) is an observation that extends the “quasi-stationary” description of the dynamics.

## Discussion

We showed in this study that the transient nature of chaotic spiral wave dynamics is not a feature of generic models but is rather a robust phenomenon which can also be found in simulations using human cardiac ion channel models (Bueno-Orovio-Cherry-Fenton model and the Ten Tusscher-Noble-Noble-Panfilov model). The general understanding of cardiac arrhythmias like ventricular fibrillation, whose electrical wave dynamics is determined by spiral or scroll waves, could benefit from the perspective of chaotic transients and its properties: Whereas the number of spiral waves grows linearly with the spatial domain size in both models, the average transient lifetime of the chaotic episodes (which would have a significant relevance when interpreted in terms of the length of cardiac arrhythmias) increases exponentially with the domain size (which might be interpreted as the heart muscle volume). In fact, the role of a critical heart muscle volume which is necessary for sustained arrhythmias has been discussed and also investigated in experimental studies [28, 29]. Also, clinical studies show, that the risk for arrhythmias and related mortality and morbidity is increased with a larger heart muscle volume [30–32].

Although the scroll wave dynamics during fibrillation of the ventricle is three-dimensional, former studies have shown that the transient features of the spiral wave chaos are also present in the three-dimensional case [16, 33]. However, in order to estimate and understand the role of transient spiral/scroll wave dynamics in the context of cardiac arrhythmias, the heterogeneous structure of the cardiac tissue and more realistic heart geometries should be taken into account in future numerical studies.

Novel control methods [34] which aim at reducing the severe side effects of conventional defibrillation methods, could benefit from an enhanced understanding of the governing mechanisms which underlie cardiac arrhythmias and self-termination of its chaotic dynamics.

Furthermore, although chaotic transients of systems characterized by an exponential increase of the average lifetime with the spatial domain size (denoted by type-II supertransients) generally terminate spontaneously and without any obvious indications a significant amount of time before the collapse, we showed that in the specific case of chaotic spiral wave dynamics the wave breakup behavior can have a significant impact on the final phase of an episode. In simulations of the BOCF model we observed that before self-termination the system remains in a relatively stable single-spiral state. This behavior coincides with the clinical observation that recorded self-termination of ventricular fibrillation in human patients showed an intermediate state of higher organization in the electrocardiogram [35, 36], e.g. ventricular tachycardia which may be associated with a single rotor. Observing this effect in both, numerical simulations and real hearts, can also be relevant regarding the question what kind of wave breakup behavior predominates during ventricular fibrillation in real hearts (specifically concerning the bidirectional transition between ventricular fibrillation and ventricular tachycardia). This knowledge can be essential when numerical simulations of cardiac tissue should reproduce the electrical wave propagation in a real heart in the most accurate way, and an appropriate models and parameters are required.

Also, in terms of a possible prediction of self-termination, statistically the absolute number of spiral waves present in the system equal to one can in this model serve as an observable indicating a higher chance for an upcoming self-termination.

From the point of view of nonlinear dynamics, the Markov chain description of the dynamics together with the significantly pronounced single-spiral state in the case of the BOCF model extends the prevailing description of type-II supertransient dynamics as “quasi-stationary”. This observation might contribute to the challenge of predicting an upcoming self-termination in these systems and the development of related applications in the field of cardiac arrhythmias.

## Supporting information

S1 Fig The temporal evolution of the number of spiral waves *N*_spiral_ during an episode of transient chaos of the BOCF model. During this episode, the system enters the “single-spiral” state (around *t* ≈ −60 *s*) but through spiral wave breakup *N*_spiral_ increases again (around *t* ≈ −50 *s*) before it finally self-terminates (at *t* = 0 *s*).
(EPS)

S1 Video Exemplary episode of the creation of initial conditions. The temporal evolution of the rescaled membrane potential *u* of a simulation of the BOCF model is shown, during the application of multiple pacing pulses, which were used to create initial conditions.
(MP4)

S2 Video Exemplary episode of transient chaotic spiral wave dynamics. The temporal evolution of the membrane potential *V*_m_ of a simulation of the TNNP model is shown during the final phase of a chaotic transient until self-termination.
(MP4)

S3 Video Video of the evolution of the (rescaled) membrane potential *u* during a transition from a multi-spiral state into the single-spiral state of the episode shown in S1 Fig. The shown episode corresponds to the episode around *t* ≈ −60 *s* of the initial condition discussed in S1 Fig.
(MP4)

S4 Video Video of the evolution of the (rescaled) membrane potential *u* during the transition from the single-spiral state into a multi-spiral state of the episode shown in S1 Fig. The shown episode corresponds to the episode around *t* ≈ −50 *s* of the initial condition discussed in S1 Fig.
(MP4)

S1 Appendix Technical details of simulations and analyzing algorithms of the data. Further information of the creation of initial conditions, the detection of the end of chaotic episodes and the determination of phase singularities are given.
(PDF)

S1 Data Numerical data underlying Figs 3, 4, 5, 6, and 7. Each folder contains data sets for the TNNP and the BOCF model, respectively.
(GZ) Click here for additional data file.
