# Supplementary material for: Spontaneous termination of chaotic spiral wave dynamics in human cardiac ion channel models
Source: PLoS One. 2019 Aug 28;14(8):e0221401. doi: 10.1371/journal.pone.0221401 (PMC6713330; doi:10.1371/journal.pone.0221401)
Supplement: S1 Appendix — Further information of the creation of initial conditions, the detection of the end of chaotic episodes and the determination of phase singularities are given. (PDF) [file pone.0221401.s006.pdf]

Appendix

Further information on the creation of initial conditions, the detection of the end of chaotic episodes and the determination of phase singularities are given in this document.

Initial-condition generator

In order to derive statistically robust statements, many independent initial conditions are required. For this purpose, we used a procedure which perturbs the dynamical variables at various spatial positions of the grid with a varying number of stimuli. It is noteworthy, that for the determination of quantities like the average transient lifetime $\langle T \rangle$, many initial conditions are needed, the exact way of how these initial conditions are constructed is, however, not relevant as long it provides spiral wave chaos.

The perturbations were injected as an external current in the case of V_m (I_stim in Eq. (1) of the main manuscript), and in a similar way for the other variables. For each initial condition, a number of pacing pulses was selected (between 10 and 15 pulses) with a varying pacing period (BOCF: 50-100 ms, TNNP: 20-120 ms). Each pulse stimulus consisted of a number of localized, square shaped pacing sites (with an edge length of $\approx 0.025\ L_x$ (BOCF) and $\approx 0.006\ L_x$ (TNNP), with L_x being the length of the simulation domain in x direction). The number of these localized pacing sites varied between $50 - 250$ (BOCF) and $500 - 800$ (TNNP). The spatial positions of the single pacing sites were homogeneously distributed over the entire simulation domain, with the only limitation that two perturbation sites do not overlap. With the choice of these parameters, the described pacing protocol could create sufficiently many independent initial conditions exhibiting spiral wave chaos.

To avoid numerical instabilities, only the membrane potential V_m was perturbed in the case of the TNNP model. We chose a single spiral wave here as an initial state (in contrast to the resting state in the case of the BOCF model) before starting the algorithm.

After the application of the perturbation pulses, a period of 1.5 s was discarded (in order to avoid correlations of the initial condition with the pulse sequence) and the resulting state was used as an initial condition for further investigations.

Figure 1 of this document shows a typical output of this implementation for the BOCF model.

Detecting the end of chaotic dynamics

The end of the chaotic dynamics involves the absence of any spiral wave in the system, and the following global decrease of the membrane potential V_m (u in the case of the BOCF model) to the resting state. We therefore determined the end of the chaotic episode by a sufficiently low spatial membrane potential variance of $\epsilon = 0.01$:

$$\frac{1}{N_x \cdot N_y} \sum_{i,j} (V_\mathrm{m}^{ij} - \langle V_\mathrm{m} \rangle)^2 < \epsilon \,, \qquad (1)$$

where N_x and N_y are the number of grid points in x and y direction, respectively, and $\langle V_\mathrm{m} \rangle$ denotes the spatial mean value of the membrane potential V_m. This choice of the threshold value was made after repeated tests involving manual inspection with both models.

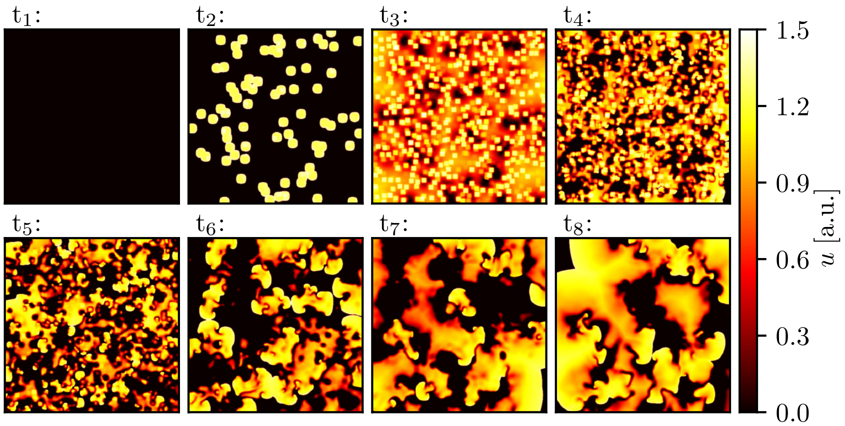

Fig 1. An exemplary creation of an initial condition in the case of the BOCF model. Snapshots of the (rescaled) membrane potential u are shown during the pulse sequence used for the creation of initial conditions. The temporal distance between two snapshots is ≈ 290 ms. An exemplary video of the creation of an initial condition is also given as supporting information (S1 Video).

Detection of phase singularities

The detection of the phase singularities was performed via a two-step protocol. In a first step, the phase θ was associated to each point of the spatial domain, by defining angles in a two-dimensional phase-space projection. It was determined by choosing two state variables X and Y via:

$$\theta = \mathrm{atan2}(Y - Y_0, X - X_0) \tag{2}$$

where X_0 and Y_0 are two reference points, and atan2 provides the angle in the euclidean plane. Table 1 lists the state variables as well as their reference values used for both models, respectively.

**Model**	X	X_0	Y	Y_0
BOCF	u	0.8 a.u.	S	0.35 a.u.
TNNP	h	0.4 a.u.	$V_\mathrm{m}^\mathrm{norm}$	0.1 a.u.

Table 1. Reference points used for the determination of the phase θ. The variable names adhere to their respective model's original nomenclature. Due to normalization, the TNNP reference value for Y is dimensionless (see Eq. (3)).

Figure 2 of this document shows a snapshot of the membrane potential u (subplot (a)) for an exemplary episode of transient spiral wave chaos (BOCF model), together with the corresponding phase θ (subplot (b)), which was calculated regarding Eq. (2).

It should be noted that the V_m variable used in the case of the TNNP model, was normalized according to

$$V_\mathrm{m}^\mathrm{norm} = \frac{V_\mathrm{m} + 86.052\ \mathrm{mV}}{112.144\ \mathrm{mV}} \tag{3}$$

prior to the subtraction of the dimensionless reference value.

In a second step, closed line integrals were evaluated at each location of the simulation domain (along a rectangle of 3×3 grid points)

$$n_\mathrm{w} = \frac{1}{2\,\pi} \oint \nabla\theta \cdot \mathrm{d}\mathbf{l}\,, \tag{4}$$

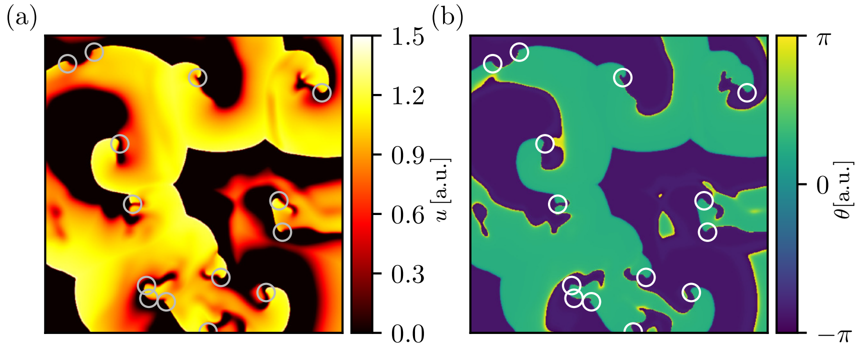

Fig 2. The detection of phase singularities. An exemplary snapshot of the (rescaled) membrane potential u of spiral wave chaos of the BOCF model (a) and the corresponding phase θ (b). In both subplots, circles indicate positions of phase singularities, which were detected via Eq. (4).

where a phase singularity was identified inside the rectangle encompassed by the line integral if $|n_\mathrm{w}| \geq 0.99$. In Fig 2 phase singularities which were determined by this method are indicated by white circles in the phase map (subplot (b)) and in the corresponding snapshot of the (rescaled) membrane potential u (gray circles in subplot (a)).

Average number of spiral waves associated to a domain size The average number of spiral waves $\langle N_\mathrm{spiral}\rangle_t$ associated with a domain size A was obtained by detecting phase singularities for an observation time of $T_\mathrm{obs} = 80$ s for each domain size and model configuration. For large domain sizes, the lifetime of a single initial condition is (in average) larger than the lifetime of an initial condition of a small domain size. That means, in the case of large domain sizes (e.g. $A = 4.96 \times 10^5\,\mathrm{mm}^2$ in the case of the BOCF model) a single initial condition with a lifetime > 80 s could be found easily. For small domain sizes, however, the transient lifetimes of single initial conditions are rather short. That is why the entire relevant time series of the total observation time T_obs is concatenated out of several time series originating from distinct initial conditions. The average $\langle N_\mathrm{spiral}\rangle_t$ (the subscript t indicates that the average was taken over time) and the standard deviation which are shown in Fig 4 of the main manuscript are then determined based on the accumulated observation time series.

Temporal evolution of average number of spiral waves during the final phase In Fig 7 of the main manuscript, the temporal evolution of the number of spiral waves during the final phase before self-termination is shown. For this purpose, the number of spiral waves of 200 initial conditions were normalized in time, such that self-termination occurs at $t = 0\,s$. The subsequent average, was then taken over the initial conditions (and not over time, as was done in the paragraph before). That is why the quantity shown in Fig 7 is denoted as $\langle N_\mathrm{spiral}\rangle_\mathrm{IC}$, where the subscript IC indicates that the average was taken over initial conditions.
